# Antitumor effect of *Melaleuca alternifolia* essential oil and its main component terpinen-4-ol in combination with target therapy in melanoma models

**DOI:** 10.1038/s41420-021-00510-3

**Published:** 2021-05-31

**Authors:** Marta Di Martile, Stefania Garzoli, Manuela Sabatino, Elisabetta Valentini, Simona D’Aguanno, Rino Ragno, Donatella Del Bufalo

**Affiliations:** 1 Preclinical Models and New Therapeutic Agents Unit, IRCCS Regina Elena National Cancer Institute, Via Elio Chianesi 53, Rome, Italy; 2 Rome Center for Molecular Design, Department of Drug Chemistry and Technology, Sapienza University, Piazzale Aldo Moro 5, Rome, Italy; 3 Department of Chemistry and Technologies of Drugs, Sapienza University, Piazzale Aldo Moro 5, Rome, Italy

**Keywords:** Melanoma, Targeted therapies

## Abstract

Essential oils (EOs) have been recently emerging for their promising biological activities in preventing tumorigenesis or progression of different tumor histotypes, including melanoma. In this study, we investigated the antitumor activity of a panel of EOs in different tumor models. The ability of *Melaleuca alternifolia* (tea tree oil) and its main component, terpinen-4-ol, to sensitize the target therapy currently used for melanoma treatment was also assessed. Our results demonstrated that EOs differently affect the viability of human cancer cells and led us to select six EOs effective in melanoma and lung cancer cells, without toxic effects in human fibroblasts. When combined with dabrafenib and/or trametinib, *Melaleuca alternifolia* synergistically reduced the viability of melanoma cells by activating apoptosis. Through machine learning classification modeling, α-terpineol, tepinolene, and terpinen-4-ol, three components of *Melaleuca alternifolia*, were identified as the most likely relevant components responsible for the EO’s antitumor effect. Among them, terpinen-4-ol was recognized as the *Melaleuca alternifolia* component responsible for its antitumor and proapoptotic activity. Overall, our study holds promise for further analysis of EOs as new anticancer agents and supports the rationale for their use to improve target therapy response in melanoma.

## Introduction

Cutaneous melanoma is the most aggressive type of skin cancer. BRAF represents the most common driver mutation present in ~50% of patients and predicting a more aggressive behavior^1^. Although target therapy and immunotherapy represent a great opportunity for melanoma treatment, patients often face lack of clinical response, the emergence of resistance to treatment, and invalidating side effects^2^. Consequently, innovative and combined therapies are still urgent to treat and eventually eradicate advanced melanoma. In light of this consideration, a large number of preclinical and clinical trials are ongoing to identify new therapeutic approaches.

Over the past decades, compounds extracted from plants have demonstrated their effectiveness in different diseases, including melanoma^3^. Examples include vinblastine^4^, vincristine^5^, paclitaxel^6^, and camptothecin^7^. Scientific evidences have demonstrated that, among natural compounds, essential oils (EOs) showed great potential for the management of a number of diseases including cardiovascular^8^, diabetes^9^, and Alzheimer^10^. EOs also represent a valid source to prevent the invasion of SARS-CoV-2 into the human body^11^, or to downregulate angiotensin-converting enzyme 2 expression in epithelial cells^12^.

Due to their minimal cytotoxicity^13,14^, EOs are considered pharmaceutically safe and could represent a good alternative natural source of anticancer agents, thus deserving further investigations to ascertain their mechanism of action and to validate their possible clinical uses as alternative/complementary antitumor agents. In the last 20 years, preclinical studies demonstrated anticancer activity of either some EOs or their main components^15,16^ and led to case–control studies^17^ and clinical trials^18–20^. At present, EOs are used to ameliorate cancer patients’ quality of life and clinical trials are ongoing to evaluate their efficacy or the efficacy of their components in cancer patients (NCT02336087, NCT03449303, NCT04560114, NCT04449315, NCT00003219, NCT00003238, NCT01459172, NCT01046929, NCT04296266). From the hundreds of studies published in the last years, it is evident that, in addition to their chemopreventive effects, several EOs and their constituents show antioxidant, antiproliferative, proapoptotic, antiangiogenic, and antimetastatic activity in melanoma models^21–23^. Synergistic effect of EO components such as geraniol^24,25^, β-elemene^26,27^, β-caryophyllene^28^, limonene^29^, eugenol^30^, and thymoquinone^31,32^ with cancer therapy has been also reported.

To shed light on the use of EOs as possible anticancer agents, in this investigation we reported the in vitro anticancer effect of a panel of EOs and investigated the possible use of *Melaleuca alternifolia* (TTO, EO05 in this investigation) as a sensitizer of targeted therapy in melanoma models. Furthermore, machine learning (ML) classification models were developed and used to investigate the possible efficacy of the more important EOs’ single components.

## Results

### A panel of EOs differently affects the viability of melanoma cells

**Fig. 1:**
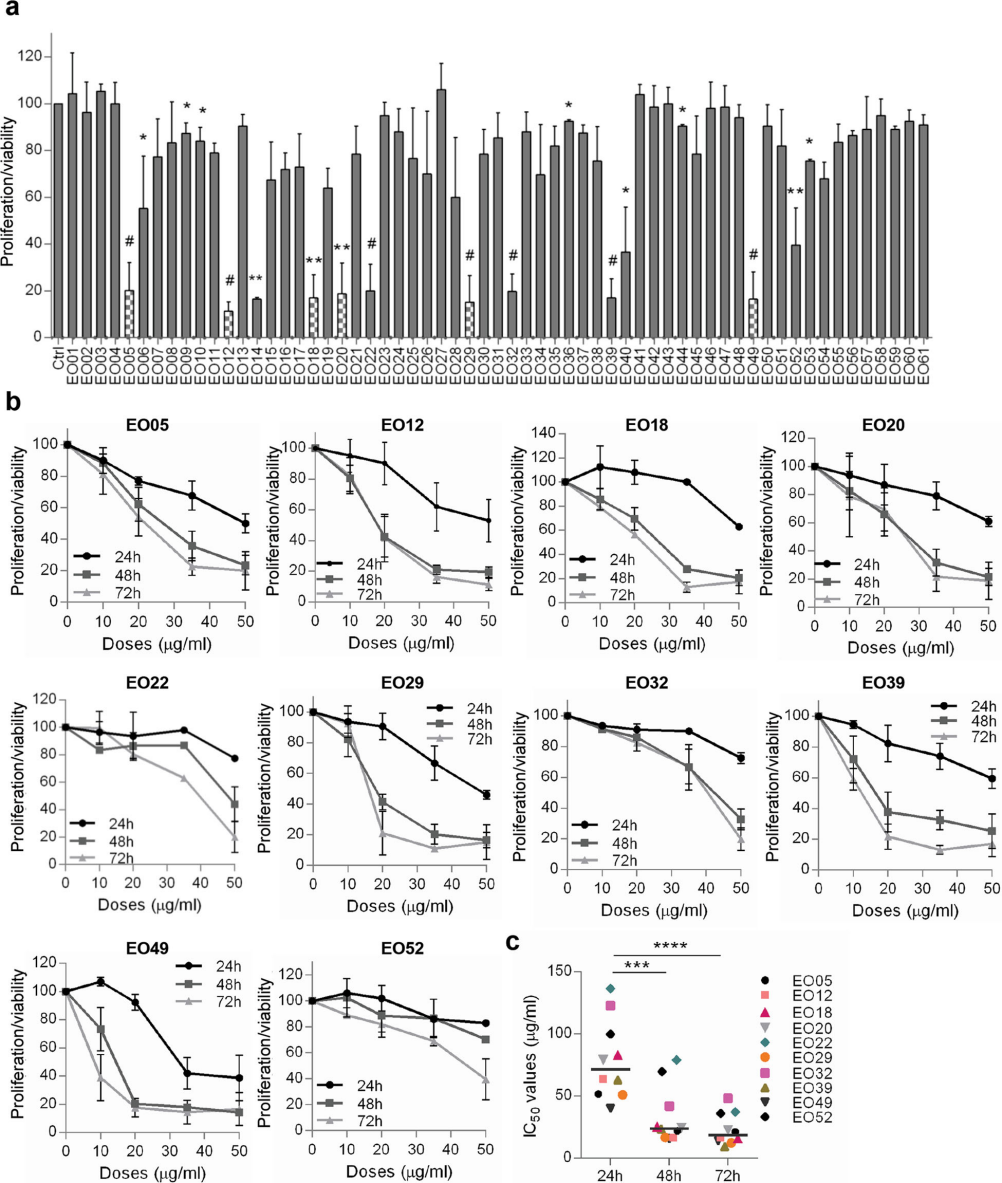
M14 cells are differentially sensitive to a panel of EOs. **a** Analysis of cell proliferation/viability by MTT assay of M14 cells treated with 61 essential oils (EOs, EO01-EO61, 50 μg/ml, 72 h). *p-*values were calculated between control (Ctrl) and EO-treated cells. *p < 0.05; **p < 0.01; ^#^*p* < 0.001 after applying Student’s *t* test. Dotted columns represent the six EOs further investigated in this study. **b** MTT assay of M14 cells treated with the indicated EOs (10–50 μg/ml, 24–72 h). **a**, **b** Results are reported as “cell proliferation-viability of treated cells/cell proliferation-viability of control cells × 100” and represent the average±standard deviation of at least three independent experiments. **c** Quantification of 50% inhibition of cell proliferation/viability (IC_50_) of the indicated EOs calculated for M14 cells treated as reported in **b**. The median of IC_50_ is shown. ***p < 0.001; ****p < 0.0001 after applying one-way ANOVA test.

The antitumor activity of 61 EOs (Table S1) was firstly assessed for their ability to affect the proliferation/viability of M14 melanoma cell line (50 μg/ml, 72 h). As reported in Fig. 1a, 18 EOs significantly reduced the proliferation/viability of M14 cells, and 12 of them inhibited at least 50% of cell proliferation. Among the 12 EOs, EO14 and EO40 were excluded from further investigations owing to their low solubility. M14 cells were treated with the remaining 10 EOs (10–50 μg/ml, 24–72 h). After 24 h treatment, a dose-dependent reduction of cell proliferation/viability was observed for seven EOs, whereas between 48 h and 72 h no significant differences in terms of IC_50_ were observed (Fig. 1b, c, Table S2). EO22, EO32, and EO52 were the less effective in reducing the M14 proliferation/viability and showing the highest deviation from the median IC_50_ for each time point (Fig. 1b, c).

As reported in Fig. S1a, the six most effective EOs (EO05, EO12, EO18, EO20, EO29, EO49), but not EO39, showed no significant effect on the proliferation/viability of normal human fibroblasts (50 μg/ml, 72 h), therefore EO39 was not further investigated. The antitumor activity of the final selected EOs was then explored on cell lines with three different tumor histotypes: lung (H1299, A549), colon (HCT116), and breast (MDA-MB-231) carcinoma. As shown in Fig. S1b, lung cancer cells treated with each EO (50 μg/ml, 48 h) were as sensitive as M14 cells, with cell proliferation/viability inhibition ranging from 67% to 82% for both cell lines used. On the contrary, the proliferation/viability of MDA-MB-231 cells was significantly reduced only by EO12, whereas HCT116 cells were resistant to the six EOs.

**Fig. 2:**
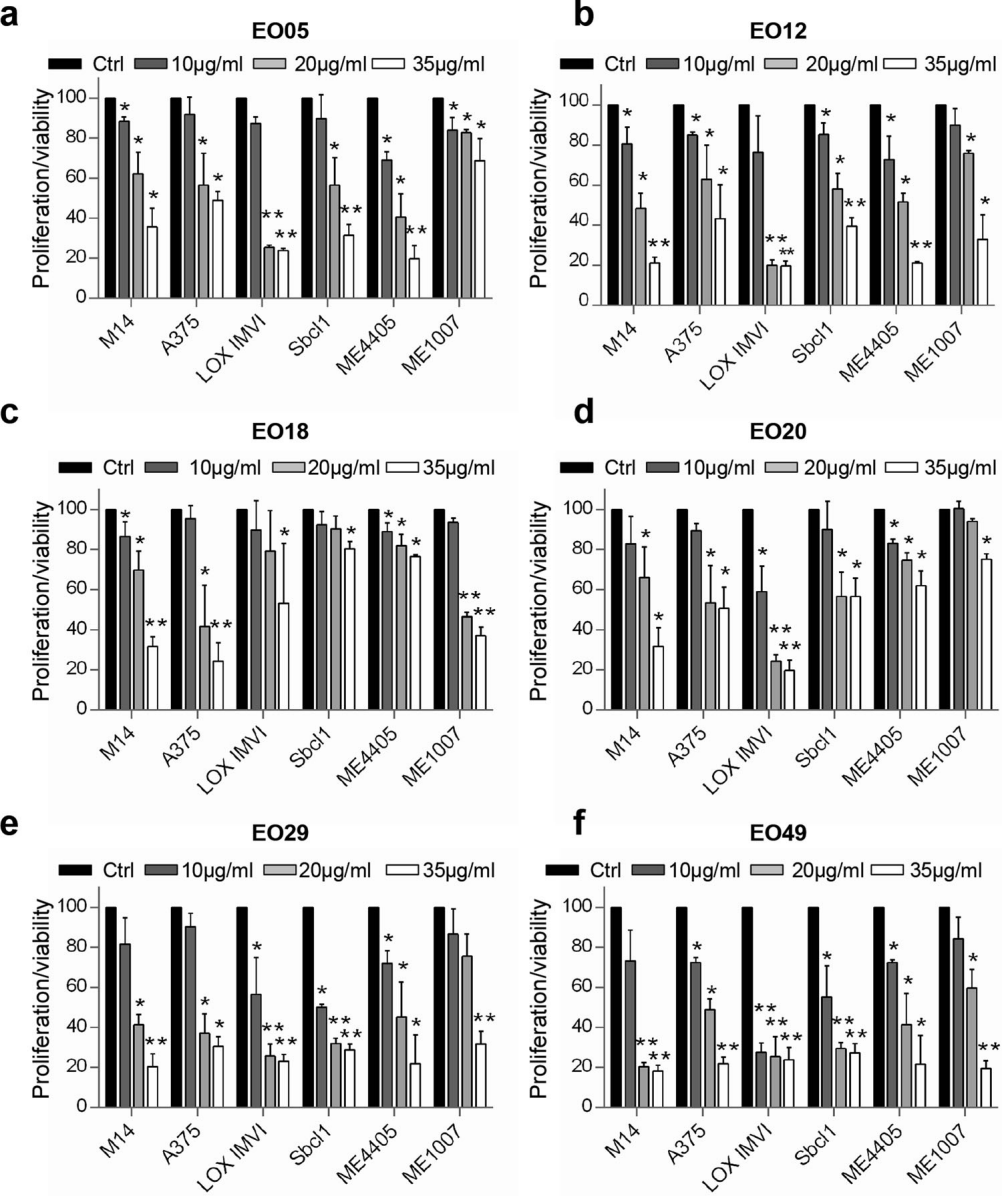
Six selected EOs affect melanoma cell proliferation/viability. **a**–**f** Analysis of cell viability by MTT assay of six melanoma cell lines treated with the indicated EOs (10–35 μg/ml, 48 h). The results are reported as “cell proliferation-viability of treated cells/cell proliferation-viability of control cells (Ctrl) × 100” and represent the average±standard deviation of at least three independent experiments. *p*-values were calculated between control and EOs treated cells. *p < 0.05; **p < 0.01 after applying Student’s *t* test.

Even though at different extend, increasing concentrations of each EO displayed a similar ability in significantly reducing the viability of both BRAF *wild type/*NRAS mutant (Sbcl1, ME4405), BRAF *wild type/*NRAS *wild type* (ME1007), and BRAF mutant/NRAS *wild type* (M14, A375, LOX IMVI) melanoma cells (Fig. 2a–f, Fig. S1c), thus indicating the absence of relevance of BRAF or NRAS status in the sensitivity to EOs.

### ML binary classification algorithms identify the most likely relevant components of EOs

To identify the most important chemical components likely responsible for viability inhibition of M14 cells, ML models were developed as reported in supplementary methods. At 50% proliferation/viability inhibition threshold, Matthews correlation coefficient and area under the curve value were 0.604 and 0.537, respectively (Fig. S2a). Inspection of the weighted feature importance values revealed α-terpineol, terpinolene, and terpinen-4-ol as those components mainly responsible for proliferation/viability inhibition of M14 cell line (Fig. S2a). The chemical composition of the EOs with the higher efficacy is reported in Table 1 and Tables S4–S8. All the three components identified through ML analysis were evidenced only in EO05 and EO49, even if at different concentrations (Table S3).

**Table 1:** Chemical composition of EO05.

No.	Component^a^	LRI^b^	LRI^c^	EO05 (%)^d^
1	α-pinene	1019	1021	11.1
2	β-pinene	1100	1105	2.5
3	β-myrcene	1157	1157	0.2
4	α-terpinene	1180	1186	4.6
5	Limonene	1195	1198	2.0
6	Eucalyptol	1201	1209	14.9
7	γ-terpinene	1236	1241	11.8
8	Terpinolene	1281	1282	1.7
9	o-cymene	1283	1287	3.5
10	Linalool oxide	1420	1423	0.2
11	α-gurjunene	1529	1527	0.2
12	Longifolene	1579	1583	0.2
13	Terpinen-4-ol	1599	1603	37.5
14	α-terpineol	1677	1675	8.1
15	Viridiflorene	1699	1695	1.1
16	Globulol	2092	2086	0.4
	Total identified			100.0

The chemical composition of EO05 was identified by GC-MS analysis.

^a^The components are reported according to their elution order on polar column.

^b^Linear Retention indices (LRI) measured on polar column.

^c^LRI from literature.

^d^Percentage mean values of EO05 components.

### EO05 sensitizes melanoma cells to target therapy

**Fig. 3:**
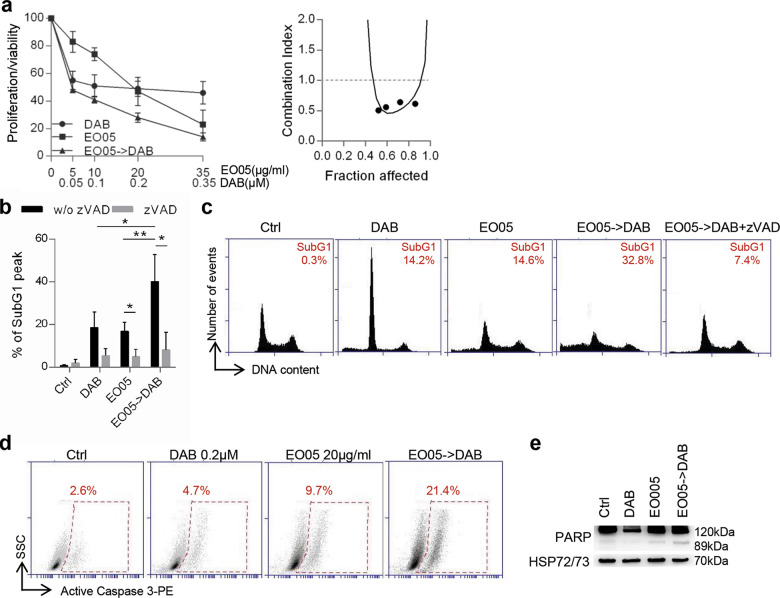
EO05 sensitizes M14 melanoma cells to dabrafenib treatment. **a** Analysis of cell proliferation/viability by MTT assay (left) and relative isobologram (right) of M14 cells after treatment with dabrafenib (DAB) or EO05 alone or 24 h EO05 followed by 48 h dabrafenib (EO05-> DAB). The results are reported as “cell proliferation-viability of treated cells/cell proliferation-viability of control cells (Ctrl) × 100”. **b** Quantification and **c** representative images of subG1 peak by propidium iodide staining of M14 cells treated with DAB (48 h, 0.2 μM), EO05 (24 h, 20 μg/ml) or with 24 h EO05 followed by 48 h dabrafenib (EO05- > DAB) in the presence or absence of zVAD (50 μM). The percentage of cells in the subG1 peak is reported. **a**, **b** The results represent the average±standard deviation of three independent experiments. Experiments with zVAD were repeated twice. **b** *p*-values were calculated between cells treated with combination and cells treated with single drugs, or between cells treated or not treated with zVAD. *p < 0.05; **p < 0.01 after applying Student’s *t* test. **d** Flow cytometric analysis of active caspase 3-PE staining in cells treated with DAB (48 h, 0.2 μM), EO05 (24 h, 20 μg/ml), or with 24 h EO05 followed by 48 h dabrafenib (EO05- > DAB). **e** Western Blot analysis of PARP cleavage in M14 cells treated as reported in **d**. HSP72/73 was used as loading and transferring control. Western blot representative of two blots with similar results is shown.

We next combined EO05, a very well characterized EO from *Melaleuca alternifolia*^33^ containing all the three components identified through the ML approach, with the targeted therapy currently used for the treatment of advanced melanoma patients harboring BRAF mutations^34^. Growth inhibitory curve and relative analysis of drug interaction demonstrated that 24 h EO05 followed by 48 h dabrafenib (BRAF inhibitor) resulted in a synergistic effect on M14 proliferation/viability reduction with combination index (CI) = 0.6 (Fig. 3a). Accordingly, this combination produced a synergistic effect also in A375 cells (Fig. S3a).

A mean of 18.5% and 16.8% of subG1 peak, indicative of dead cells, was detected after treatment with dabrafenib or EO05, respectively. Interestingly, in cells treated with EO05 followed by dabrafenib, the subG1 population significantly increased up to 40.2% (Fig. 3b, c). In addition, treatment with the caspase inhibitor zVAD-FMK (zVAD) significantly reduced the subG1 peak in cells treated with EO05 alone (4.9%) or in combination with dabrafenib (8%), thus demonstrating apoptotic cell death. Apoptosis induction was also confirmed by the increase of active caspase 3 and cleaved PARP in cells treated with the combination when compared to single treatments (Fig. 3d, e).

**Fig. 4:**
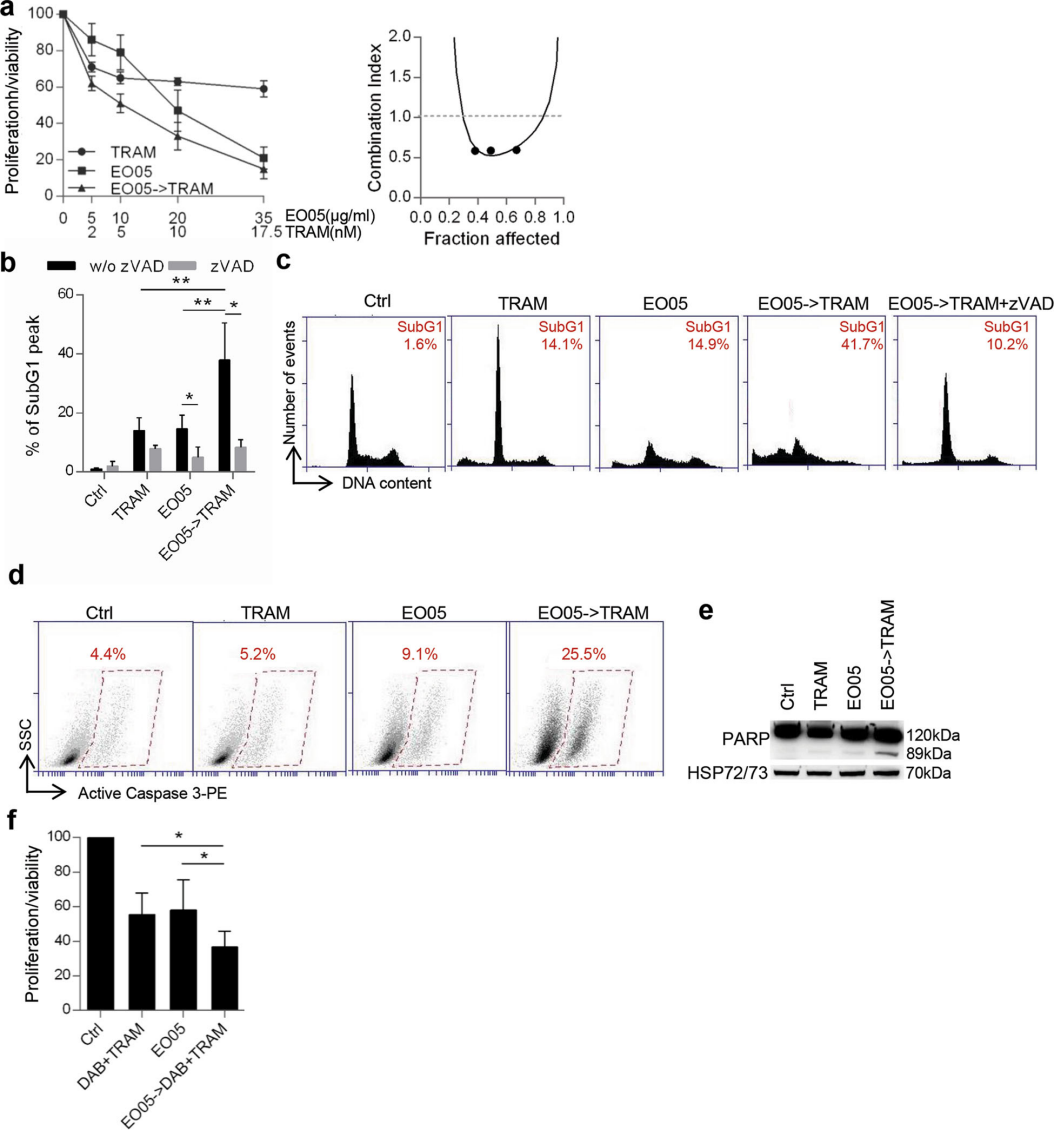
EO05 sensitizes M14 melanoma cells to trametinib treatment. **a** Analysis of cell proliferation/viability by MTT assay (left) and relative isobologram (right) of M14 cells treated with 48 h trametinib (TRAM) or 24 h EO05 alone or 24 h EO05 followed by 48 h trametinib (EO05- > TRAM). **b** Quantification and **c** representative images relative of subG1 peak by propidium iodide staining of M14 cells control (Ctrl) or treated with TRAM (48 h, 10 nM), EO05 (24 h, 20 μg/ml) or with 24 h EO05 followed by 48 h TRAM (EO05- > TRAM), in the presence or absence of zVAD (50 μM). The percentage of cells in the subG1 peak is reported. **d** Flow cytometric analysis of active caspase 3-PE staining in cells treated with TRAM (48 h, 10 nM), EO05 (24 h, 20 μg/ml), or with 24 h EO05 followed by 48 h TRAM (EO05- > TRAM). **e** Western blot analysis of PARP cleavage in M14 cells treated as reported in **d**. HSP72/73 was used as loading and transferring control. Western blot representative of two blots with similar results is shown. **f** MTT assay of M14 cells treated with dabrafenib (0.001 μM)+trametinib (0.1 nM) for 48 h, EO05 (20 μg/ml) for 24 h alone or 24 h EO05 followed by 48 h DAB + TRAM (EO05- > DAB + TRAM). **a**, **f** The results are reported as “cell proliferation-viability of treated cells/cell proliferation-viability of control cells × 100”. **a**, **b**, **f** The results represent the average ± standard deviation of three independent experiments. Experiments with zVAD were repeated twice. **b**, **f** *p*-values were calculated between cells treated in combination and cells treated with single drugs, or between cells treated or not treated with zVAD. *p < 0.05; **p < 0.01 after applying Student’s *t* test.

Similar to what observed for dabrafenib, administration of 24 h EO05 followed by 48 h trametinib (MEK inhibitor) showed a synergistic effect strongly reducing M14 cell proliferation/viability (CI = 0.5) (Fig. 4a). Accordingly, treatment of EO05 followed by trametinib increased the percentage of subG1 peak, caspase 3, and PARP cleavage (Fig. 4b–e) when compared with trametinib or EO05 alone. Moreover, the addition of zVAD significantly decreased the subG1 peak in cells treated with EO05 alone or in combination (Fig. 4b, c). A synergistic effect of EO05 followed by trametinib was also obtained in the BRAF *wild type* melanoma cells, ME4405 (CI = 0.6) (Fig. S3b). Next, the effect of EO05 in combination with dabrafenib and trametinib, the current standard treatment for BRAF mutant melanoma patients, was also assessed. Interestingly, 24 h EO05 followed by 48 h of dabrafenib/trametinib treatment strongly reduced the proliferation/viability of M14 cells compared with exposure to EO05 alone or to dabrafenib/trametinib (Fig. 4f).

### Terpinen-4-ol is responsible for EO05 antitumor activity

**Fig. 5:**
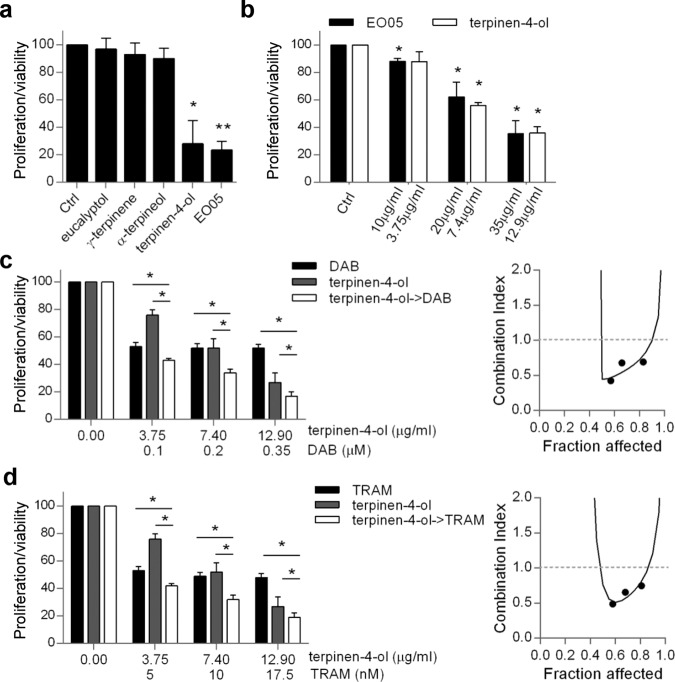
Terpinen-4-ol is responsible for EO05 antitumor activity in M14 cells. **a** MTT assay of M14 cells treated for 72 h with eucalyptol (7 μg/ml), γ-terpinene (6 μg/ml), α-terpineol (4 μg/ml), terpinen-4-ol (18.5 μg/ml) or EO05 (50 μg/ml). **b** MTT assay of M14 cells treated with the indicated concentrations of EO05 or of terpinen-4-ol. **c**, **d** MTT assay (left) and relative isobologram (right) of M14 cells treated with **c** dabrafenib (DAB), **d** trametinib (TRAM), or terpinen-4-ol alone or in combination (24 h terpinen-4-ol followed by 48 h DAB or TRAM). **a**–**d** The results are reported as “cell proliferation-viability of treated cells/cell proliferation-viability of control cells × 100”. The results represent the average±standard deviation of at least three independent experiments. *p*-values were calculated between control (Ctrl) and treated cells or cells treated in combination and cells treated with single drugs. *p < 0.05; **p < 0.01, after applying Student’s *t* test.

Four among the most abundant components of EO05, identified by gas chromatography mass spectroscopy (GC/MS) analysis (Table 1) were tested for their ability to affect M14 and A375 cell proliferation/viability at the concentration contained in 50 μg/ml of EO05. Terpinen-4-ol (18.5 μg/ml, 48 h), was the only component that significantly reduced M14 (Fig. 5a) and A375 (Fig. S4a) proliferation/viability of ~70% and 60%, respectively, an effect similiar to that exerted by EO05 at 50 μg/ml. On the contrary, eucalyptol (7 μg/ml), γ-terpinene (6 μg/ml), and α-terpineol (4 μg/ml) had no significant effect on M14 and A375 cell proliferation/viability (Fig. 5a, Fig. S4a). Furthermore, treatment with terpinen-4-ol for 48 h significantly decreased M14 (Fig. 5b) and A375 (Fig. S4b) cell proliferation/viability in a dose-dependent manner, up to 64% and 56%, respectively, likewise EO05 (64.3% for M14 and 51% for A375, respectively).

**Fig. 6:**
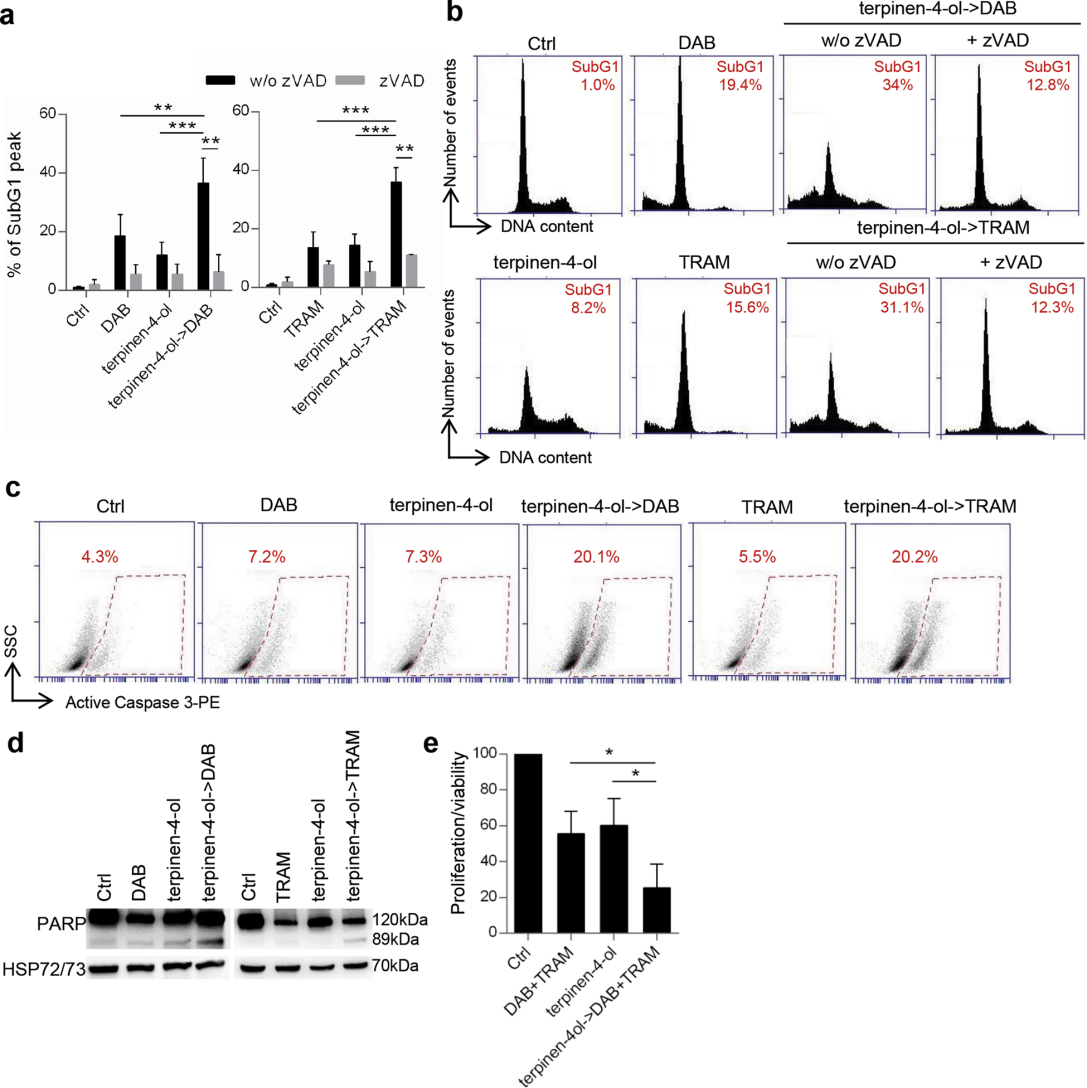
Terpinen-4-ol induces apoptosis in combination with targeted therapy. **a** Quantification and **b** representative images relative of subG1 peak by propidium iodide staining of M14 cells treated with 48 h dabrafenib (DAB, 0.2 μM) or trametinib (TRAM, 10 nM), 24 h terpinen-4-ol (7.4 μg/ml) alone or in combination (24 h terpinen-4ol->48 h DAB/TRAM), in the presence or absence of zVAD (50 μM). **c** Flow cytometric analysis of active caspase 3-PE staining in cells treated with 48 h dabrafenib (0.2 μM) or trametinib (10 nM), 24 h terpinen-4-ol (7.4 μg/ml) alone or in combination (24 h terpinen-4ol->48 h DAB/TRAM). **d** Western blot analysis of PARP cleavage in M14 cells treated as reported in **c**. HSP72/73 was used as loading and transferring control. Western blot representative of two blots is shown. **e** MTT assay of M14 cells treated with dabrafenib (0.001 μM)+trametinib (0.1 nM) for 48 h, terpinen-4-ol (7.4 μg/ml) for 24 h alone or 24 h terpinen-4-ol followed by 48 h DAB + TRAM (terpinen-4-ol->DAB + TRAM). The results are reported as “cell proliferation-viability of treated cells/cell proliferation-viability of control cells × 100”. **a**, **e** The results represent the average±standard deviation of three independent experiments. Experiments with zVAD were repeated twice. *p*-values were calculated between control (Ctrl) and treated cells, cells treated in combination and cells treated with single drugs, or between cells treated or not treated with zVAD. *p < 0.05; **p < 0.01, ***p < 0.001 after applying Student’s *t* test.

Interestingly, as determined for EO05, terpinen-4-ol pre-treatment synergistically reduced cell viability of M14 cell line when associated with dabrafenib (CI = 0.44) (Fig. 5c) or trametinib (CI = 0.7) (Fig. 5d). Accordingly, an increased subG1 peak, reduced by the addition of zVAD, was observed in cells treated with combinations with respect to single treatments (Fig. 6a, b). The apoptotic induction of the combinations was confirmed by the increase of PARP and caspase 3 cleavage (Fig. 6c, d). Analogous results were obtained for A375 when terpinen-4-ol was followed by dabrafenib (CI = 0.5) or trametinib (CI = 0.47) (Fig. S4c, d). Interestingly, the terpinen-4-ol pre-treatment strongly synergized the effect of dabrafenib/trametinib treatment (Fig. 6e).

## Discussion

In this study, we provided evidence about the antiproliferative effect of a panel of EOs in melanoma and lung carcinoma cells. More importantly, we determined the ability of TTO to synergize with target therapy in melanoma models. In particular, an initial screening of 61 EOs led to select six of them (TTO, *Pinus Sylvestris, Lavandula Angustifolia, Citrus Paradisi, Pinus Sibirica, Cupressus Sempervirens*) as the most efficacious in terms of reduction of tumor cell proliferation/viability, without affecting normal fibroblasts viability. We also found that the efficacy of EOs depends on the tumor histotype examined. In fact, the treatment with the six EOs reduced cell proliferation of melanoma and lung carcinoma cells in a dose-dependent manner, whereas they were ineffective in breast and colon carcinoma cells. The mechanism that renders the different histotypes differently sensitive to the six EOs is not yet clear. No reports have been yet published about the six EOs used in colon cancer models. Nevertheless, TTO has been reported to induce apoptosis in breast cancer cells at concentration six times higher than those we used in our study^35^, whereas *Pinus Sylvestris* EO (EO29) exhibited some potential as an antiproliferative agent in the same cellular model (i.e., MDA-MB-231)^36^, thus suggesting a different composition of EO used. In fact, we and other authors previously reported that multiple factors affect EO composition^37–40^.

A panel of melanoma cell lines, harboring *wild type* or mutant BRAF and NRAS, showed sensitivity to the six EOs, even if at a different extend, thus indicating that the effect of EOs was not related to BRAF or NRAS status.

All the six selected EOs, except for *Pinus Sibirica (*EO20*)*, were investigated for their effect on cancer^41–45^ but only TTO (EO05) showed antitumor efficacy in preclinical melanoma models. In particular, through its most abundant component, terpinen-4-ol, TTO has been reported to reduce cell proliferation^46–48^, cause cell cycle perturbation^47,48^, induce necrosis^47^ or apoptosis^46,48^, and interfere with in vitro invasive/migratory capability^49^ of melanoma models. Moreover, a topical formulation of TTO retarded the in vivo growth of subcutaneous melanoma and evidenced immune effector cell recruitment on the treated region^50^. Considering all these effects, the EOs lipophilicity, the fact that EOs are well absorbed through the skin^51^, as well as the fact that chemoprevention is an essential approach for cancer control^52^, TTO has been suggested as a possible chemopreventive candidate to be used in topical formulations against melanoma and other types of skin cancer^48,53^.

Despite the great interest in TTO reported in the last years^54,55^, the contribution of TTO as a sensitizer of cancer, and in particular, of melanoma therapy^56,57^, is unknown. We demonstrated that TTO synergized with dabrafenib and trametinib, when administered either as single agents or in combination, in terms of apoptosis induction, when TTO treatment was followed by exposure to one of the two drugs. However, we cannot exclude that TTO alone and in combination with targeted therapy may activate other forms of cell death.

In agreement with studies demonstrating that among TTO components, terpinen-4-ol is responsible of TTO efficacy^46,47,49^, we demonstrated the relevance of terpinen-4-ol the main component present in TTO (37.5%), in the antiproliferative effect and in the sensitization to target therapy. In mouse or human melanoma cells, TTO and terpinen-4-ol elicited G1 cell cycle arrest, showed an antiproliferative effect, antimigratory/antiinvasive ability against cells resistant to chemotherapy, and induced necrotic and apoptotic cell death^46,47,49^. We and other authors also reported terpinen-4-ol ability to affect in vitro and in vivo growth of tumors with different origin^58–62^, and to enhance the effect of several chemotherapeutic or biological agents in cancers not including melanoma^61^. Results from ML analysis performed on the M14 screening were in good agreement with experimental data effectively indicating terpinen-4-ol as one of the components mainly responsible for viability inhibition of melanoma cells. Indeed, among the final selected six EOs, EO05 did contain terpinen-4-ol at the highest percentage. The antiproliferative effect of EO12, EO18, EO20, EO29, and EO49, showing low or non-detectable levels of terpinen-4-ol could be due to other components present in their composition and reported to affect proliferation of melanoma cells, such as linalool^63^, limonene^64^, camphene^65^, α-, and β-pinene^66^.

In agreement with studies demonstrating (i) the nature of terpenes as lipophilic molecules able to disrupt normal structure and function of cell membranes^46^, and (ii) the ability of TTO and terpinen-4-ol to interact with the lipid bilayer of cellular membranes and to inhibit the intracellular signaling induced by p170 glycoprotein^49,67^, we suggest that the synergistic effect of TTO or terpinen-4-ol with target therapy could be related to their effect on plasma membrane, i.e., reorganization of lipid architecture, thus favoring the entrance of drug in the cell.

Our data are in agreement with previous studies reporting the ability of EOs such as *Cymbopogon citratus*, or EO components, such as β-elemene and thymoquinone, to increase the efficacy of radiation in melanoma models^68,69^, or curcumol, β-caryophyllene, citral, or valencene to enhance the sensitivity of tumors from different origin to antineoplastic treatment^70–72^.

To the best of our knowledge, this is the first study examining the ability of TTO, and in particular, terpinen-4-ol, to potentiate the targeted therapy of melanoma, highlighting the importance of our investigation. The efficacy of the combination TTO/target therapy could be of relevant importance as it can lead to the use of a lower concentration of drugs commonly used for the management of melanoma patients and consequently lower toxic treatments in terms of side-effect and more efficacious. The potential use of TTO is further supported by its non-toxicity in normal cells^35^ and by its penetrability in the skin^73^.

Supported by low toxicity and side-effect of EOs, as well as their good tolerance by patients, our study hold promise for further analysis of EOs as new anticancer drugs and/or as a source of potential anticancer supplement against melanoma. The effect of TTO on melanoma cells and the analysis of its main components are worthy of further investigation.

## Materials and methods

### Cell cultures

Human melanoma (M14, A375, LOX IMVI, Sbcl1, ME4405, and ME1007) and lung cancer (H1299, A549) cell lines were cultured in Roswell Park Memorial Institute 1640 medium (Euroclone, Milan, IT). Colon cancer (HCT116), breast cancer (MDA-MB-231) cells, and human telomerase reverse transcriptase immortalized fibroblasts (BJ-hTERT) were cultured in Dulbecco’s Modified Eagle’s medium (Lonza, Basilea, CH) supplemented with 10% inactivated bovine serum (Gibco, Thermo Fisher Scientific, MA, USA). ME4405 and ME1007 cell lines were established as reported^74^. Sbcl1 cell line was provided by Beppino G Giovannella^75^. All the other cell lines were purchased from American Type Culture Collection (Manassas, VA). Cells were routinely tested for mycoplasma contamination and were recently authenticated.

### Reagents preparation and treatment

EOs (Farmalabor srl, Assago, IT), dabrafenib, trametinib (Selleckchem Chemicals, Houston, TX, USA) and zVAD (abcam, Cambridge, UK) were dissolved in dimethyl sulfoxide (DMSO, Sigma Aldrich, St. Louis, MO, USA) and further diluted in complete medium. Cells were treated up to 0.001% DMSO as vehicle control. Eucalyptol, γ-terpinene, α-terpineol, and terpinen-4-ol were diluted in complete medium. Methanol (Sigma Aldrich) was used to dilute EOs for GC-MS analysis.

### Analysis of cell proliferation/viability

In all, 3 × 10^3^ cells/well were seeded in 96-well plates and treated for 24–72 h. Cell proliferation**/**viability was evaluated by measuring 3-[4,5-dimethylthiazol-2-yl]-2,5-diphenyltetrazolium bromide inner salt (MTT, Sigma Aldrich) dye absorbance as previously reported^76^. The concentration of drug that reduces 50% of cell viability (IC_50_) and CI were analyzed by using median-effect method (Calcusyn software, Biosoft). CI values of <1, =1, and >1 indicate, respectively, synergistic, additive, and antagonistic effects.

### Western blot and flow cytometric analyses

Western blot analyses were performed as previously reported^77^ using primary antibodies directed to PARP (cod. 51-6639GR, BD Bioscience, San Jose, CA) or HSP72/73 (cod. D00175805, Calbiochem, Saint Diego, CA, USA,) as control of loading and transfer. Anti-mouse immunoglobulin G-horseradish peroxidase-conjugated antibody (cod. 1858413, Amersham Biosciences, Freiburg, Germany) was used as a secondary antibody.

Cell cycle distribution by propidium iodide staining was performed as previously described^78^. Caspase 3 activation was evaluated using an active caspase 3-PE antibody (cat. 559565, BD Bioscience, San Jose, CA), following the manufacturer’s instructions. All the cytofluorimetric analyses were performed using BD Accuri^TM^ C6 flow cytometer.

### GC-MS analysis

GC-MS analyses were carried out using a Perkin Elmer Clarus 500 GC equipped with a flame ionization detector and coupled with a Clarus 500 mass spectrometer. A Stabilwax capillary column (Restek, Bellefonte, PA, USA) was used with helium as carrier gas (1.0 mL/min). GC oven temperature was kept at 60°C for 5 min and programmed to 220°C at a rate of 5°C/min, and kept constant at 220°C for 30 min. Mass spectra were acquired over 40–500 amu with ionizing electron energy 70 eV. In all, 1 μL of the EO was diluted in 1 mL of methanol and 1 μL of the solution was injected into the GC injector at 280°C. The identification of compounds of EOs was performed by comparing mass spectra with those reported in Nist and Wiley libraries. Linear retention indices were calculated after injection of C8–C30 aliphatic hydrocarbons mixture under the same conditions described above and compared with available linear retention indices data in the literature.

### ML binary classification

All calculations were performed using the Python programming language (version 3.7, https://www.python.org/) by executing in-house code in the Jupyter Notebook platform, as previously reported^79,80^. For details see supplementary material and Table S9, 10.

### Statistics

Unless otherwise indicated, at least three independent experiments have been performed. Six technical points for each experimental group were used for MTT assay. The data were expressed as mean ± standard deviation or ± standard error of the mean. For continuous variables, differences between two groups were analyzed with Student’s *t* test (unpaired, two-sided). One-way ANOVA test was used to analyze differences between the three groups. *P* < 0.05 was considered statistically significant. All statistical tests and the estimation of variation between groups were performed with GraphPad Prism 6 (GraphPad Software, Inc., La Jolla, CA, USA). All data were included in the analyses. Based on the variation shown in our preliminary results, we determined the sample sizes by using power analysis.

## Supplementary information

Supplementary Material

Fig. S1

Fig. S2

Fig. S3

Fig. S4

## Data Availability

Data sets related to this article can be found at [https://gbox.garr.it/garrbox/index.php/s/R8CXBDawomyk632].
