# Supplementary material for: Antitumor effect of Melaleuca alternifolia essential oil and its main component terpinen-4-ol in combination with target therapy in melanoma models
Source: Cell Death Discov. 2021 May 31;7:127. doi: 10.1038/s41420-021-00510-3 (PMC8165351; doi:10.1038/s41420-021-00510-3)
Supplement: Supplementary file 1 — Supplementary Material [file 41420_2021_510_MOESM1_ESM.docx]

**SUPPLEMENTARY MATERIAL**

**Machine Learning (ML) Binary Classification**

As similarly reported^1,2^, all calculations were performed using the Python programming language (version 3.7, <https://www.python.org/>) by executing in-house code in the Jupyter Notebook platform. The biological data and essential oil chemical composition were imported and loaded into a Python Pandas dataframe and pre-processed to the final datasets to develop the classification models. ML algorithms used in this study were implemented using the Scikit-learn library (sklearn)^3^. Unsupervised dimensionality reduction was performed with principal component analysis (PCA)^4^ to extract 85% of explained variance. Cross-validation (CV) was used to evaluate the robustness of the final models as well as during the hyperparameters’ tuning. Different cut-off values were used to obtain the optimized hyperparameters classification models for each strain. A first hyperparameters selection was achieved through 3000 randomized runs from all possible considered combinations^5^. Furthermore, variables which take only a few values (nlevels) and, in addition, have ill distribution of the objects in these levels were pruned out of the table. Nlevel variables are dangerous as they force the under training model to fit most of the variance of a few objects with a high leverage, thus leading to spurious and misleading results. Column pruning was applied up to 4 unique levels. A final optimization was completed through a systematic variation (grid search) of the numerical random selected hyperparameters varying the values in a range of ± 10. To develop the models, different linear and non-linear ML classification algorithms were used: random forest (RF), gradient bosting (GB), support vector (SV), logistic regression (LR), decision tree (DT), and *k* nearest neighbors (KNN) as implemented in sklearn. The binary classification models were numerically and graphically evaluated by accuracy (ACC), F1 score, Matthews correlation coefficient (MCC), receiver operating characteristic (ROC) and area under the curve score (AUC). The importance of EOs chemical components was individually evaluated through the “feature importance” (FI) and partial dependence (PD)^6^ as implemented in the Skater python library^7,8^. Internal models’ validation was carried out by leave-some-out CV using 5 groups by means of the stratified K-fold method while monitoring the average value of MCC obtained from 50 random CV iterations ^9,10^. Final models were selected based on both MCC and ROC AUC values.

**Datasets**

Considering the proliferation/viability of M14 cells (**Figure 1a**), the initial dataset was loaded into a Pandas dataframe. Each dataset was composed by a data matrix of 61 rows (essential oil samples) and 243 columns (one bioactivity and 242 chemical components). To evaluate the under developing ML model ability in discriminating M14 cell proliferation/viability modulating EOs, the biological data were binarized using a 50% threshold value.

**Classification Models**

Classification models were built with a number of latent variables corresponding to 85% of the whole chemical components variance extracted by PCA. Hyperparameter optimization was carried out with a wide range of settings (**Table S9**), leading from hundreds of thousands to billions of combinations. Therefore, to speedup the optimization, the random search was used. Random search hyperparamenters’ optimization was proved, having a probability of 95% of finding a combination of parameters within the 5% optima with only 60 iterations, while reducing the probability to bog down in local optima^11^. Accordingly, herein 3000 random combinations were used at four different nlevels and, as a last step, the models from random search were refined by a grid search inspecting numerical hyperparameters in a range of ± 10. Thus, the models after the last systematic grid search tuning were pruned on the basis of MCC and AUC cutoff values set to 0.6 and 0.5^12,13,14,15^, respectively. As a result, among the six ML algorithms, DT algorithm led to define statistically acceptable models (**Table S10**). FIs were inspected to investigate the most important chemical components likely responsible 50% viability inhibition. Moreover, PDs were finally investigated to seek for the statistical responsibility for each model’s most important chemical components.

**Chemical Components Importance and Partial Dependences**

Chemical component importance was evaluated through FIs and PDs. Each FI indicates a sort of absolute correlation coefficient for each of the chemical components, while the associated PD gives its negative, positive or neutral influence. Therefore, PDs positive or negative trends were investigated by means of a spearman correlation (SP) coefficient, which is known to range from −1 to 1. The SP values were used to positively or negatively weight the corresponding FI values to obtain positive or negative weighted FIs (WFIs). WFIs were inspected by means of bar plots in a straightforward interpretation. For sake of clarity and redundancy avoidance, the analysis was focused on the top 20 FI values).

**Supplementary Tables**

**Table S1**. Identification (ID) and plant name of essential oils used in the investigation

| **EO ID** | **Plant Name** | **EO ID** | **Plant Name** |
| --- | --- | --- | --- |
| EO1 | Chamomile Morocco | EO32 | Birch |
| EO2 | Sage Sclarea | EO33 | Fennel |
| EO3 | Salvia Officinalis | EO34 | Cedar Fruit |
| EO4 | Red Thyme | EO35 | Lemon |
| EO5 | Tea Tree Oil (*Melaleuca Alternifolia*) | EO36 | Roman Chamomile |
| EO6 | Melissa Oiio Essential | EO37 | Savory |
| EO7 | Pinus Mugo | EO38 | Rosemary |
| EO8 | Geranium Bourbon | EO39 | Ceylon Cinnamon Peel |
| EO9 | Oregano | EO40 | Eucaliptus Globulus |
| EO10 | Ylang Ylang | EO41 | Sweet Orange |
| EO11 | Coriander | EO42 | Niaouly |
| EO12 | Lavandula Angustifoglia | EO43 | Artemisia |
| EO13 | Myrtle | EO44 | Cajeput |
| EO14 | Garlic | EO45 | Black Pepper |
| EO15 | Cardamom | EO46 | White Thyme |
| EO16 | Mandarin | EO47 | Marjoram |
| EO17 | Hyssop | EO48 | Cloves |
| EO18 | Grapefruit (*Citrus Paradisi*) | EO49 | Cypress (*Cupressus Sempervirens*) |
| EO19 | Cymbopogon | EO50 | Nutmeg Natural |
| EO20 | Pinus Sibirica | EO51 | Peppermint |
| EO21 | Camphor | EO52 | Verbena officinalis |
| EO22 | Cadè | EO53 | Basil |
| EO23 | Cedar Leaves | EO54 | Cymbopogon martinii |
| EO24 | Ginger | EO55 | Laurel |
| EO25 | Cumin | EO56 | Anise |
| EO26 | Patchouli | EO57 | Incense |
| EO27 | Bitter Orange | EO58 | Mentha Suaveolens |
| EO28 | Eucalyptus | EO59 | Coridotthymus Capitatus |
| EO29 | Pinus Sylvestris | EO60 | Thymus Vulgaris |
| EO30 | Bergamot | EO61 | Origanum Hirtum |
| EO31 | Juniper |  |  |

The scientific names of the most investigated plants from which EOs were extracted is indicated in brackets.

**Table S2.** IC_50_ values of M14 cells after 24-72h of EOs treatment

|  | **24h** | **48h** | **72h** |
| --- | --- | --- | --- |
| **EO05** | 51 μg/ml | 21 μg/ml | 21 μg/ml |
| **EO12** | 64 μg/ml | 16 μg/ml | 16 μg/ml |
| **EO18** | 83 μg/ml | 25 μg/ml | 16 μg/ml |
| **EO20** | 79 μg/ml | 24 μg/ml | 22 μg/ml |
| **EO22** | 136 μg/ml | 79 μg/ml | 37 μg/ml |
| **EO29** | 51 μg/ml | 16 μg/ml | 12 μg/ml |
| **EO32** | 122 μg/ml | 41 μg/ml | 48 μg/ml |
| **EO39** | 63 μg/ml | 23 μg/ml | 9 μg/ml |
| **EO49** | 40 μg/ml | 15 μg/ml | 13 μg/ml |
| **EO52** | 99 μg/ml | 69 μg/ml | 36 μg/ml |

**Table S3.** Comparison of the ML most important chemical components and their presence in the selected EOs

| **Chemical component** | **EO05** | **EO12** | **EO18** | **EO20** | **EO29** | **EO49** | **# non zero values**^a^ | **max**^b^ |
| --- | --- | --- | --- | --- | --- | --- | --- | --- |
| α-terpineol | 8.10 | 1.27 | 0 | 0.44 | 0.89 | 0.38 | 5 | 8.10 |
| terpinolene | 1.67 | 0 | 0 | 0 | 2.01 | 0.69 | 3 | 2.01 |
| terpinen-4-ol | 37.49 | 3.77 | 0 | 0 | 0 | 1.52 | 3 | 37.49 |

Percentage mean values of EO components (%) is reported; ^a^ occurrence of the chemical component in the six selected EOs; ^b^ max percentage of the chemical component

**Table S4.** Chemical composition of EO12

| **N°** | **COMPONENT^1^** | **LRI^2^** | **LRI ^3^** | **EO12 (%) ^4^** |
| --- | --- | --- | --- | --- |
| 1 | α-pinene | 1020 | 1021 | 0.2 |
| 2 | β-myrcene | 1152 | 1157 | 0.7 |
| 3 | limonene | 1195 | 1198 | 0.5 |
| 4 | eucalyptol | 1201 | 1209 | 2.4 |
| 5 | cis-β-ocimene | 1233 | 1237 | 1.0 |
| 5 | trans-β-ocimene | 1250 | 1252 | 1.3 |
| 6 | 3-octanone | 1271 | 1270 | 0.9 |
| 7 | 3-octanol | 1403 | 1406 | 0.3 |
| 8 | butanoic acid, hexyl ester | 1408 | 1410 | 0.5 |
| 9 | 1-octen-3-ol | 1460 | 1458 | 0.3 |
| 10 | linalool | 1535 | 1537 | 34.7 |
| 11 | linalyl acetate | 1551 | 1553 | 41.4 |
| 12 | lavandulyl acetate | 1587 | 1584 | 4.3 |
| 13 | terpinen-4-ol | 1605 | 1603 | 3.8 |
| 14 | β-caryophyllene | 1621 | 1619 | 2.7 |
| 16 | β-farnesene | 1628 | 1630 | 1.9 |
| 17 | α-terpineol | 1672 | 1675 | 1.3 |
| 18 | borneol | 1691 | 1697 | 1.4 |
| 19 | geraniol | 1805 | 1802 | 0.4 |
|  | **Total identified** |  |  | 100.0 |

^1^The components are reported according to their order on polar column; ^2^Linear Retention indices (LRI) measured on polar column; ^3^LRIs from literature; ^4^percentage mean values of components (%).

**Table S5.** Chemical composition of EO18

| **N°** | **COMPONENT^1^** | **LRI^2^** | **LRI ^3^** | **EO18**  **(%)^4^** |
| --- | --- | --- | --- | --- |
| 1 | α-pinene | 1020 | 1021 | 0.4 |
| 2 | β-myrcene | 1152 | 1157 | 0.3 |
| 3 | limonene | 1195 | 1198 | 78.2 |
| 4 | o-cymene | 1285 | 1287 | 0.3 |
| 5 | cis-limonene-oxide | 1455 | 1458 | 3.5 |
| 5 | trans-limonene-oxide | 1460 | 1466 | 2.3 |
| 6 | trans-p-mentha-2,8-dienol | 1520 | * | 2.1 |
| 7 | p-mentha-2,8-dien-1-ol | 1620 | 1622 | 1.9 |
| 8 | carvone | 1738 | 1740 | 4.7 |
| 9 | carveol | 1812 | 1816 | 1.9 |
| 10 | cis-carveol | 1865 | 1866 | 3.3 |
| 11 | caryophyllene oxide | 1887 | 1892 | 0.4 |
| 12 | limonene-1,2-diol | 2222 | 2226 | 0.7 |
|  | **Total identified** |  |  | 100.0 |

^1^The components are reported according to their order on polar column; ^2^Linear Retention indices (LRI) measured on polar column; ^3^LRIs from literature; ^*^LRI^lit^ not available; ^4^ percentage mean values of components.

**Table S6.** Chemical composition of EO20

| **N°** | **COMPONENT^1^** | **LRI^2^** | **LRI ^3^** | **EO20**  **(%)^4^** |
| --- | --- | --- | --- | --- |
| 1 | santene | 980 | 984 | 1.0 |
| 2 | tricyclene | 1000 | 1002 | 1.5 |
| 3 | α-pinene | 1020 | 1021 | 8.4 |
| 4 | camphene | 1060 | 1065 | 18.1 |
| 5 | β-pinene | 1100 | 1105 | 1.6 |
| 5 | limonene | 1195 | 1198 | 3.9 |
| 6 | β-phellandrene | 1201 | 1204 | 1.1 |
| 7 | o-cymene | 1281 | 1287 | 0.4 |
| 8 | camphor | 1500 | 1507 | 0.4 |
| 9 | bornyl acetate | 1562 | 1567 | 55.0 |
| 10 | β-caryophyllene | 1621 | 1619 | 0.9 |
| 11 | humulene | 1668 | 1667 | 0.5 |
| 12 | α-terpineol | 1672 | 1675 | 0.4 |
| 13 | crypton | 1678 | 1675 | 0.6 |
| 14 | borneol | 1692 | 1697 | 2.7 |
| 16 | β-bisabolene | 1720 | 1717 | 0.2 |
| 17 | p-cymen-8-ol | 1841 | 1838 | 0.7 |
| 18 | caryophyllene oxide | 1887 | 1892 | 1.3 |
| 19 | humulene-1,2-epoxide | 2043 | 2040 | 0.6 |
| 20 | p-cymen-7-ol | 2082 | 2078 | 0.2 |
| 21 | α-bisabolol | 2233 | 2230 | 0.4 |
|  | **Total identified** |  |  | 99.9 |

^1^The components are reported according to their order on polar column; ^2^Linear Retention indices (LRI) measured on polar column; ^3^LRIs from literature; ^4^ percentage mean values of components.

**Table S7.** Chemical composition of EO29

| **N°** | **COMPONENT^1^** | **LRI^2^** | **LRI ^3^** | **EO29**  **(%)^4^** |
| --- | --- | --- | --- | --- |
| 1 | α-pinene | 1020 | 1021 | 22.8 |
| 2 | camphene | 1060 | 1065 | 0.9 |
| 3 | β-pinene | 1100 | 1105 | 17.1 |
| 4 | 3-carene | 1142 | 1146 | 16.6 |
| 5 | limonene | 1195 | 1198 | 11.2 |
| 5 | terpinolene | 1278 | 1282 | 2.0 |
| 6 | o-cymol | 1285 | 1287 | 0.5 |
| 7 | α-cubebene | 1470 | 1475 | 0.5 |
| 8 | bornyl acetate | 1562 | 1567 | 1.4 |
| 9 | longifolene | 1890 | 1583 | 2.0 |
| 10 | β-caryophyllene | 1621 | 1619 | 16.7 |
| 11 | pinocarveol | 1655 | 1651 | 0.4 |
| 12 | verbenol | 1690 | 1652^+^ | 0.2 |
| 13 | humulene | 1671 | 1667 | 1.2 |
| 14 | α-terpineol | 1692 | 1690^+^ | 0.9 |
| 15 | myrtenol | 1799 | 1792 | 0.2 |
| 16 | p-cymen-8-ol | 1842 | 1838 | 1.6 |
| 17 | caryophyllene oxide | 1887 | 1892 | 3.5 |
| 18 | humulene-1,2-epoxide | 1900 | * | 0.3 |
|  | **Total identified** |  |  | 100.0 |

^1^ The components are reported according to their order on polar column; ^2^Linear Retention indices (LRI) measured on polar column; ^3^LRIs from literature; ^+^Normal alkane RI; *LRIlit not available; ^4^ percentage mean values of components.

**Table S8.** Chemical composition of EO49

| **N°** | **COMPONENT^1^** | **LRI^2^** | **LRI ^3^** | **EO49**  **(%)^4^** |
| --- | --- | --- | --- | --- |
| 1 | α-pinene | 1020 | 1021 | 47.7 |
| 2 | camphene | 1060 | 1074 | 0.6 |
| 3 | β-pinene | 1100 | 1105 | 1.3 |
| 4 | sabinene | 1103 | 1107 | 0.7 |
| 5 | 3-carene | 1142 | 1146 | 29.3 |
| 5 | limonene | 1195 | 1198 | 4.0 |
| 6 | eucalyptol | 1201 | 1209 | 0.5 |
| 7 | terpinolene | 1278 | 1282 | 0.7 |
| 8 | o-cymene | 1281 | 1287 | 1.4 |
| 9 | linalool | 1535 | 1537 | 0.7 |
| 10 | verbenol | 1562 | 1652^+^ | 0.3 |
| 11 | cedrene | 1588 | * | 0.5 |
| 12 | terpinen-4-ol | 1605 | 1603 | 1.5 |
| 13 | α-terpineol | 1672 | 1675 | 0.4 |
| 14 | α-terpineol acetate | 1718 | 1721 | 3.8 |
| 15 | verbenone | 1734 | 1730 | 0.3 |
| 16 | 2-caren-4-ol | 1821 | 1816^+^ | 0.4 |
| 17 | p-cymen-8-ol | 1841 | 1838 | 1.2 |
| 18 | cedrol | 2111 | 2109 | 4.7 |
|  | **Total identified** |  |  | 100.0 |

^1^ The components are reported according to their order on polar column; ^2^Linear Retention indices (LRI) measured on polar column; ^3^LRIs from literature; ^+^Normal alkane RI; ^*^LRI^lit^ not available; ^4^ percentage mean values of components.

**Table S9**. List of hyperparameters setting used for the models’ random search optimization

| **Algorithm** | **Parameters** | **Settings** | **Total Combinations** |
| --- | --- | --- | --- |
| rf | class_weight | list_weight | 133970183 |
|  | n_estimators | from 1 to 200, step 1 |  |
|  | max_depth | from 1 to 200, step 1 |  |
|  | min_samples_leaf | from 1 to 200, step 1 |  |
| gb | n_estimators | from 1 to 200, step 1 | 7880599 |
|  | max_depth | from 1 to 200, step 1 |  |
|  | min_samples_leaf | from 1 to 200, step 1 |  |
| lr | class_weight | list_weight | 50745 |
|  | C | from 1 to 200, step 1 |  |
|  | penalty | l1, l2, elasticnet |  |
|  | solver | newton-cg, lbfgs, liblinear, sag, saga |  |
|  | max_iter | 10000 |  |
| sv | class_weight | list_weight | 13532 |
|  | C | from 1 to 200, step 1 |  |
|  | kernel | linear, poly, rbf, sigmoid |  |
|  | probability | True |  |
| dt | class_weight | list_weight |  |
|  | criterion | gini, entropy | 2176000 |
|  | splitte<r | best, random |  |
|  | max_depth | from 1 to 21, step 1 |  |
|  | min_samples_split | from 1 to 21, step 1 |  |
|  | min_samples_leaf | from 1 to 21, step 1 |  |
|  | max_features | auto, sqrt, log2, None |  |
| knn | n_neighbors | from 1 to 31, step 1 | 57600 |
|  | weights | uniform, distance |  |
|  | algorithm | auto, ball_tree, kd_tree, brute |  |
|  | leaf_size | from 1 to 31, step 1 |  |
|  | metric | minkowski, euclidean, manhattan, chebyshev |  |
|  | metric_params | None |  |
|  | p | 1, 2 |  |

**Table S10.** List of machine learning (ML) model selected hyperparameter and statistical coefficients

| **Model**^a^ | **ML** |
| --- | --- |
| **Classifier**^b^ | dt |
| **Threshold %^c^** | 50 |
| **# Toxic**^d^ | 49 |
| **# Non Toxic**^e^ | 12 |
| **# comp**^f^ | 242 |
| **nlevel**^g^ | 4 |
| **# nleveled comp^h^** | 60 |
| **# PC*^i^*** | 22 |
| **Final Hyperparameter settings**^j^ | {'splitter': 'random', 'min_samples_split': 16, 'min_samples_leaf': 1, 'max_features': None, 'max_depth': 11, 'criterion': 'entropy', 'class_weight': {0: 1.2, 1: 1.0}} |
| **Accuracy**^k^ **(fitting)**^l^ | 0.89 |
| **F1**^m^ **(fitting)** | 0.63 |
| **AUC**^n^ **(fitting)** | 0.93 |
| **MCC**^o^ **(fitting)** | 0.598156113 |
| **Accuracy (crossvalidated)**^p^ | 0.89 |
| **F1 (crossvalidated)** | 0.59 |
| **AUC (crossvalidated)** | 0.54 |
| **MCC (crossvalidated)** | 0.60 |

^a^ Machine learning model id; ^b^ machine learning algorithm as defined in Material and Methods; threshold value used to binarize the M14 viability data; ^d^ number of compounds found to block viability at threshold value; ^e^ number of compounds found not to block viability at threshold value; ^f^ number of chemical components from all EOs; ^g^ value to eliminate column with nlevel number of non-zero variables; ^h^ number of chemical components after application of nlevel filtering; ^i^ number of principal components explaining 85% of variance; ^k^ accuracy score; ^l^ indicate values obtained with non-cross-validated runs; ^m^ F1 score; ^n^ ROC AUC score; ^o^ Matthew correlation coefficient; ^g^ ROC AUC values; p indicate values obtained from cross-validation.

**Supplementary Figure legends**

**Figure S1.** **Six selected EOs differently affect proliferation/viability of cancer cell lines from different histotypes.** (a) Analysis of cell viability by MTT assay of BJhTERT fibroblasts treated with the indicated essential oils (EOs) (20-50μg/ml, 72h). (b) Analysis of cell viability by MTT assay of melanoma (M14), lung (H1299, A549), colon (HCT116) and breast (MDA-MB-231) carcinoma cell lines treated with the indicated EOs (50μg/ml, 48h). (a,b) The results are reported as «cell proliferation-viability of treated cells/cell growth/viability of control cells (Ctrl) x 100» and represent the average±standard deviation of three independent experiments. p-values were calculated between control and treated cells. *p<0.05; **p<0.01; #p<0.001 by applying Student’s t-test. (c) Evaluation of the sensitivity of BRAF *wild type/*NRAS mutant (Sbcl1, ME4405), BRAF *wild type/*NRAS *wild type (*ME1007) and BRAF mutant/NRAS *wild type* (M14, A375, LOX IMVI) melanoma cell lines to the indicated EOs for 48h. The y-axis shows the EO concentration causing a 50% inhibition of cell proliferation/viability (IC_50_).

**Figure S2**. **α-terpineol, terpinolene and terpinen-4-ol are the most likely relevant components of EOs identified by Machine Learning (ML) analysis. (a)** Weighted feature importance plot for models ML obtained on the dataset binarized at 50% proliferation/viability inhibition. Positive bars are associated with proliferation/viability inhibition, whereas negative bars are associated with low non-toxic effect on viability.

**Figure S3.** **EO05 sensitizes melanoma cells to Dabrafenib and Trametinib treatment.** (a) Analysis of cell viability by MTT assay (left panel) and relative pharmacological interaction (right panel) of A375 cells treated with the indicated concentrations of Dabrafenib (DAB) or EO05 alone or the combination (24h EO05 treatment followed by 48h DAB treatment, EO05->DAB). (b) Analysis of cell viability by MTT assay (left panel) and relative pharmacological interaction (right panel) of ME4405 cells treated with the indicated concentrations of Trametinib (TRAM) or EO05 alone or the combination (24h EO05 treatment followed by 48h TRAM treatment, EO05->TRAM). (a,b) The results are reported as «proliferation-viability of treated cells/proliferation-viability of control cells x 100» and represent the average±standard deviation of three independent experiments.

**Figure S4.** **Terpinen-4-ol is responsible for EO05 antitumor activity in A375 cells.** (a) MTT assay of A375 cells treated for 72h with eucalyptol (7μg/ml), γ-terpinene (6μg/ml), α-terpineol (4μg/ml), terpinen-4-ol (18.5μg/ml) or EO05 (50μg/ml). (b) MTT assay of A375 cells treated with the indicated concentrations of EO05 or of terpinen-4-ol. (c) MTT assay (left panel) and relative pharmacological interaction (right panel) of A375 cells treated with the indicated concentrations of (c) Dabrafenib (DAB), terpinen-4-ol alone or in combination (24h terpinen-4-ol treatment followed by 48h DAB, terpinen-4-ol->DAB), (d) Trametinib (TRAM), terpinen-4-ol alone or in combination (24h terpinen-4-ol followed by 48h TRAM, terpinen-4-ol ->TRAM). (a-d) The results represent the average±standard deviation of three independent experiments. p-values were calculated (a,b) between control (Ctrl) and treated cells or (c,d) between cells treated in combination and cells treated with the single drugs. *p<0.05, **p<0.01 by applying Student’s t-test.

**References**

1. Patsilinakos A, Artini M, Papa R, Sabatino M, Bozovic M, Garzoli S*, et al.* Machine Learning Analyses on Data including Essential Oil Chemical Composition and In Vitro Experimental Antibiofilm Activities against Staphylococcus Species. *Molecules* 2019, **24**(5).

2. Papa R, Garzoli S, Vrenna G, Sabatino M, Sapienza F, Relucenti M*, et al.* Essential Oils Biofilm Modulation Activity, Chemical and Machine Learning Analysis. Application on Staphylococcus aureus Isolates from Cystic Fibrosis Patients. *Int J Mol Sci* 2020, **21**(23).

3. Pedregosa F, Varoquaux G, Gramfort A, Michel V, Thirion B, Grisel O*, et al.* Scikit-learn: Machine Learning in Python. *Journal of Machine Learning Research* 2011, **12:** 2825-2830.

4. Pechenizkiy M, Tsymbal A, Puuronen S. PCA-based feature transformation for classification: Issues in medical diagnostics. *17th Ieee Symposium on Computer-Based Medical Systems, Proceedings* 2004**:** 535-540.

5. Bergstra J, Bardenet R, Kégl B, Bengio Y. *Algorithms for Hyper-Parameter Optimization*, 2011.

6. Friedman JH. Greedy function approximation: A gradient boosting machine. *Annals of Statistics* 2001, **29**(5)**:** 1189-1232.

7. Choudhary P, Kramer A, team dc. Datascienceinc/Skater: Enable Interpretability via Rule Extraction(BRL). Zenodo; 2018.

8. Wei P, Lu Z, Song J. Variable importance analysis: A comprehensive review. *Reliability Engineering & System Safety* 2015, **142:** 399-432.

9. Baldi P, Brunak S, Chauvin Y, Andersen CAF, Nielsen H. Assessing the accuracy of prediction algorithms for classification: an overview. *Bioinformatics* 2000, **16**(5)**:** 412-424.

10. Artini M, Patsilinakos A, Papa R, Bozovic M, Sabatino M, Garzoli S*, et al.* Antimicrobial and Antibiofilm Activity and Machine Learning Classification Analysis of Essential Oils from Different Mediterranean Plants against Pseudomonas aeruginosa. *Molecules* 2018, **23**(2).

11. Zheng A. *Evaluating Machine Learning Models*. O'Reilly Media, Inc., 2015.

12. Chicco D, Jurman G. The advantages of the Matthews correlation coefficient (MCC) over F1 score and accuracy in binary classification evaluation. *BMC Genomics* 2020, **21**(1)**:** 6.

13. Halimu C, Kasem A, Newaz SHS. Empirical Comparison of Area under ROC curve (AUC) and Mathew Correlation Coefficient (MCC) for Evaluating Machine Learning Algorithms on Imbalanced Datasets for Binary Classification. *Proceedings of the 3rd International Conference on Machine Learning and Soft Computing (Icmlsc 2019)* 2019**:** 1-6.

14. Akoglu H. User's guide to correlation coefficients. *Turk J Emerg Med* 2018, **18**(3)**:** 91-93.

15. Powers D, Ailab. Evaluation: From precision, recall and F-measure to ROC, informedness, markedness & correlation. *J Mach Learn Technol* 2011, **2:** 2229-3981.
