# Supplementary figures and images for: Antitumor effect of Melaleuca alternifolia essential oil and its main component terpinen-4-ol in combination with target therapy in melanoma models
Source: Cell Death Discov. 2021 May 31;7:127. doi: 10.1038/s41420-021-00510-3 (PMC8165351; doi:10.1038/s41420-021-00510-3)

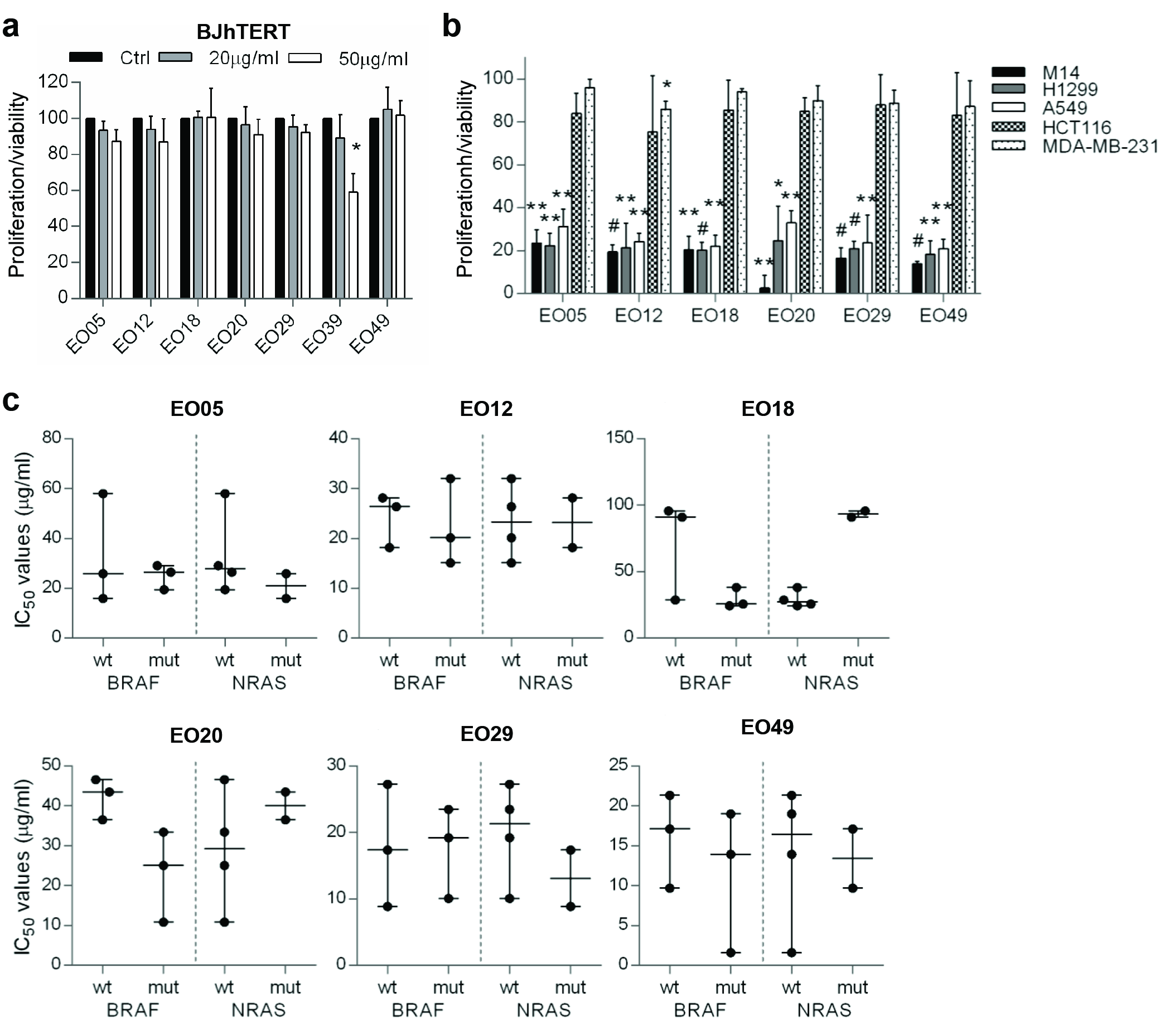

Supplement: Supplementary file 2 — Fig. S1 [file 41420_2021_510_MOESM2_ESM.tif]

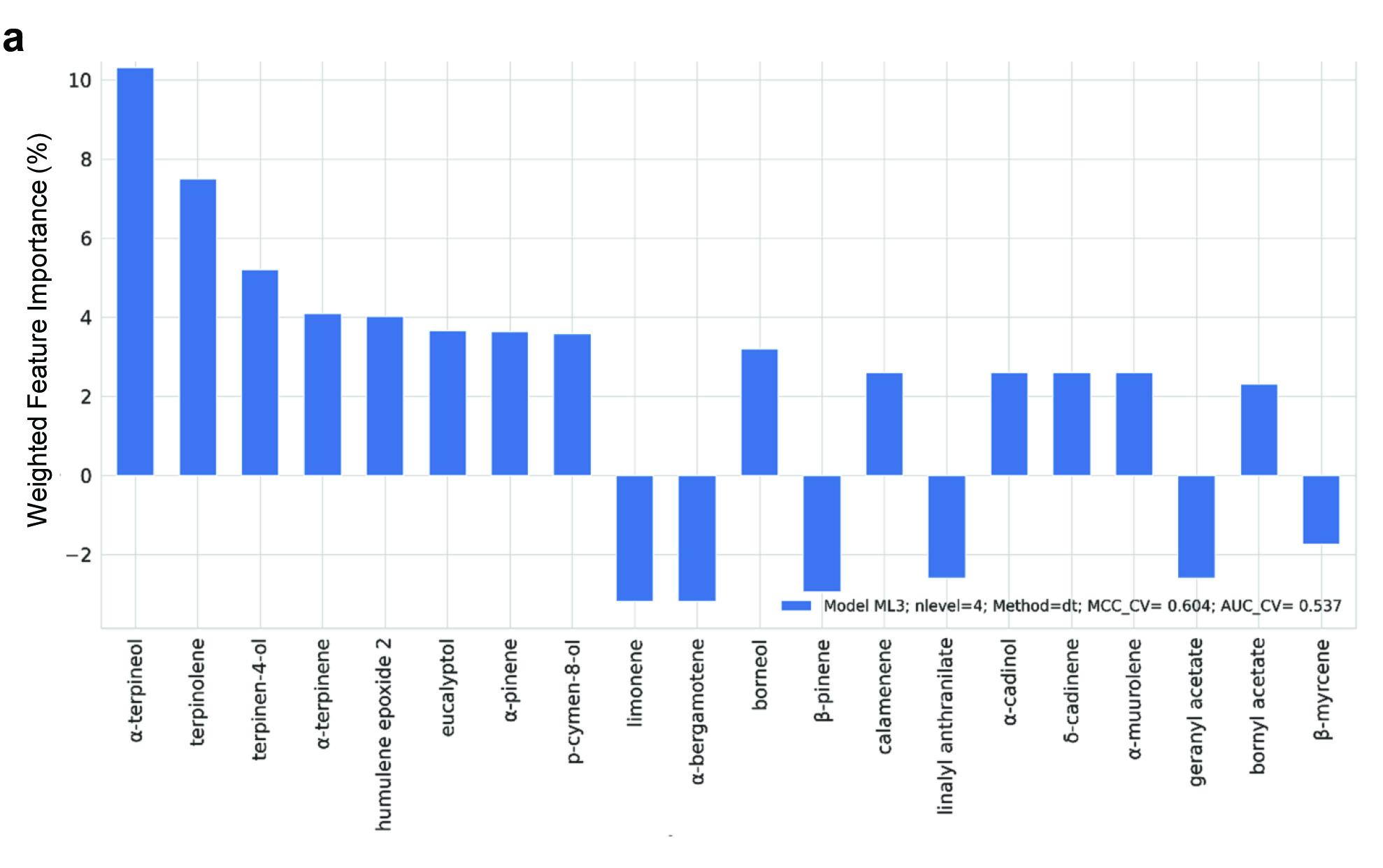

Supplement: Supplementary file 3 — Fig. S2 [file 41420_2021_510_MOESM3_ESM.tif]

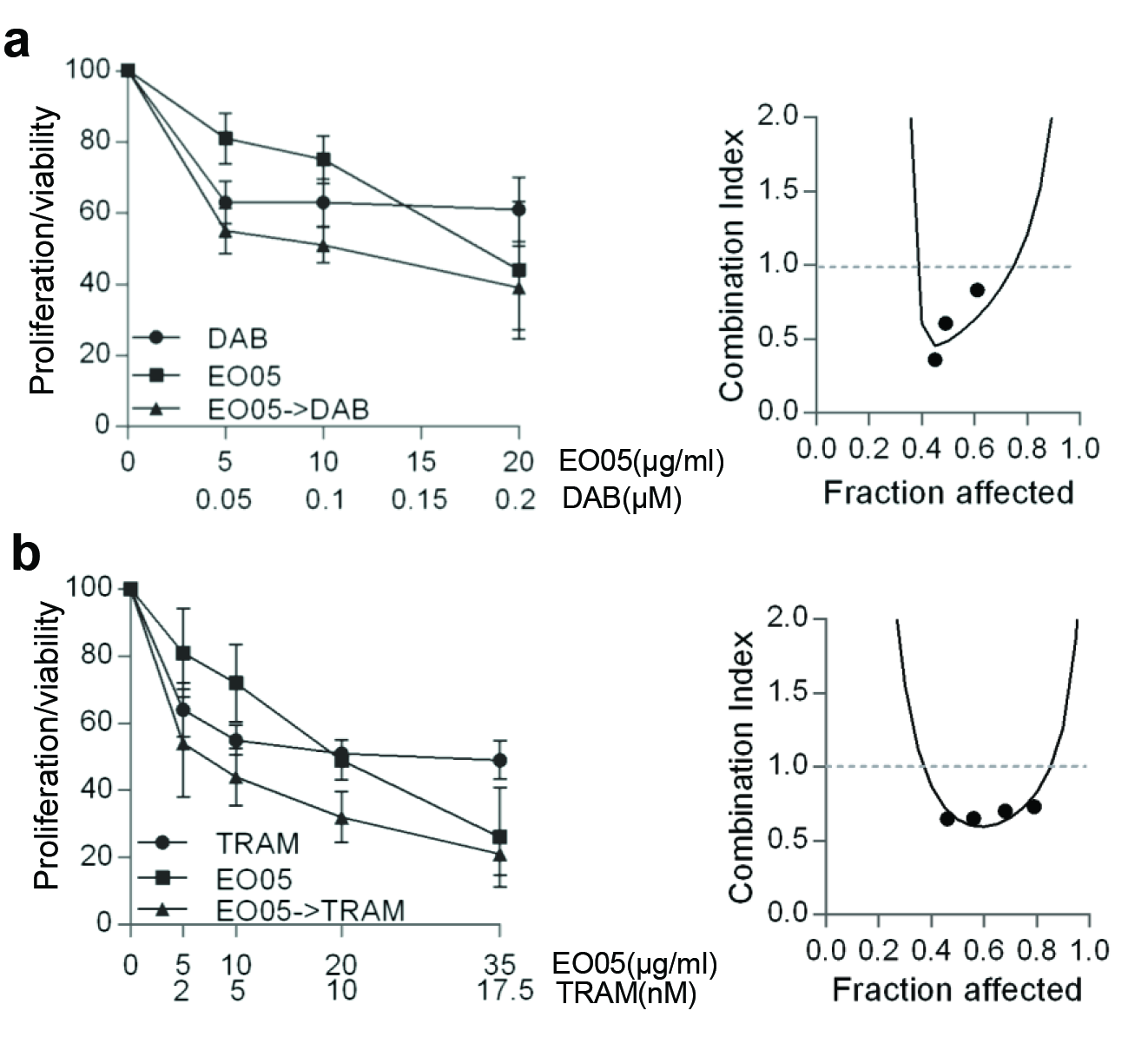

Supplement: Supplementary file 4 — Fig. S3 [file 41420_2021_510_MOESM4_ESM.tif]

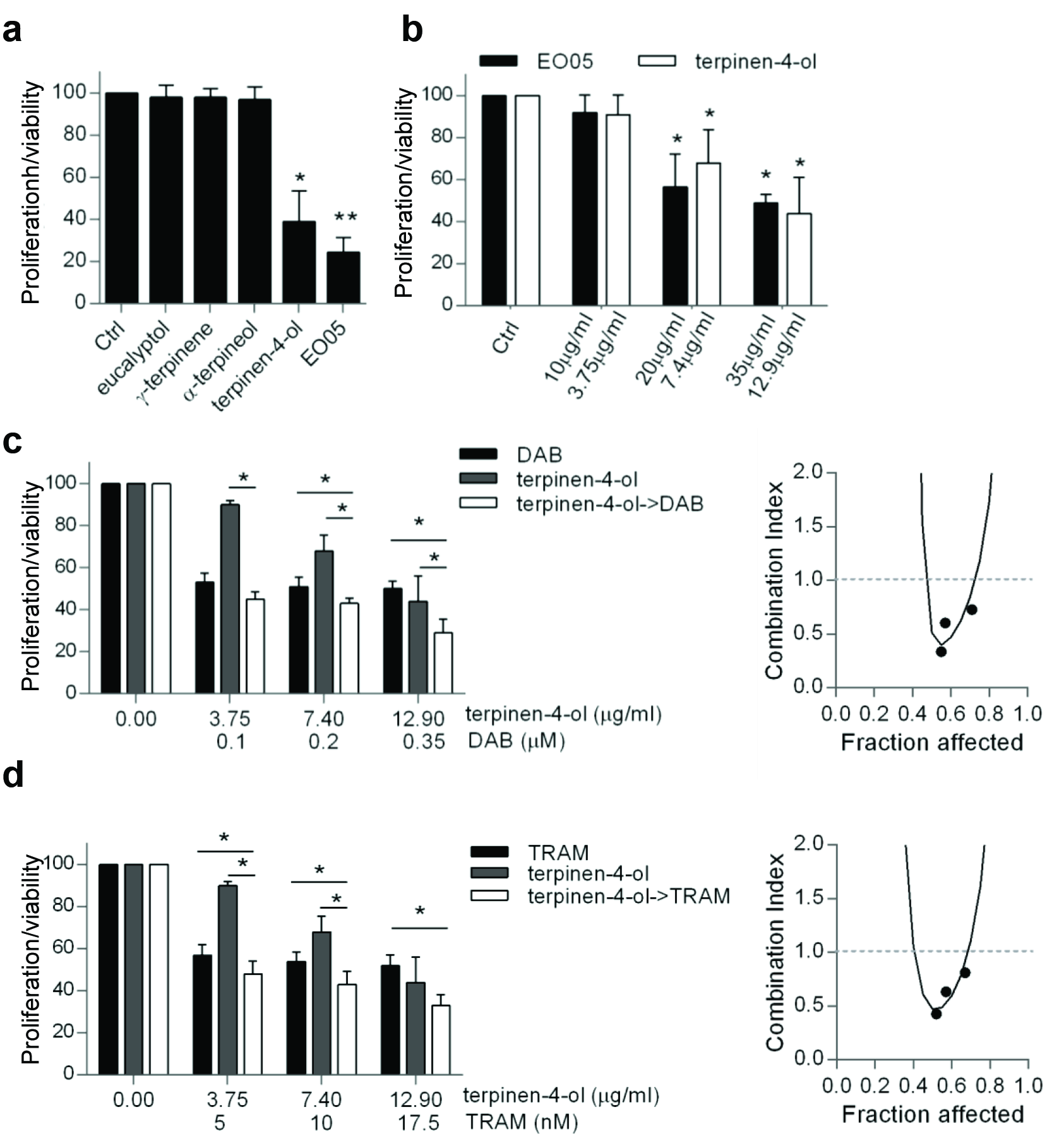

Supplement: Supplementary file 5 — Fig. S4 [file 41420_2021_510_MOESM5_ESM.tif]
